# *Staphylococcus aureus* Induces IFN-β Production via a CARMA3-Independent Mechanism

**DOI:** 10.3390/pathogens10030300

**Published:** 2021-03-04

**Authors:** Yang Zhou, Shasha Zhao, Xiao Gao, Songhong Jiang, Jialu Ma, Rui Wang, Qing Li, Leiying Qin, Zhizi Tong, Junwei Wu, Jianjun Zhao

**Affiliations:** 1 College of Veterinary Medicine, Southwest University, Chongqing 402460, China; 15087307880@139.com (S.Z.); gaoxiaoyouxiangya@163.com (X.G.); 15859788060@163.com (S.J.); mjl0698@163.com (J.M.); wr07100914@163.com (R.W.); 18335940855@163.com (L.Q.); Zhizi_tung@163.com (Z.T.); wjw999@163.com (J.W.); 2 Immunology Research Center, Medical Research Institute, Southwest University, Chongqing 402460, China; 3 College of Animal Science and Technology, Southwest University, Chongqing 402460, China; li1299616089@163.com

**Keywords:** CARMA3, IFN-β, TBK1, IRF3, *Staphylococcus aureus*

## Abstract

Type I interferon (IFN) induction is a critical component of innate immune response to viral and bacterial infection, including *S. aureus*, but whether it activates the signaling in macrophages and the regulation mechanisms is less well understood. Here we show that *S. aureus* infection promoted the IFN-β mRNA expression and stimulator of IFN genes (STING)/TANK-binding kinase 1 (TBK1)/interferon regulatory factor 3 (IRF3)-dependent production of IFN-β. Infection with *S. aureus* induced caspase recruitment domain and membrane-associated guanylate kinase-like domain protein 3 (CARMA3) expression at both the mRNA and protein levels. The heat-killed bacteria failed to trigger IRF3 phosphorylation and upregulation of CARMA3 expression. However, overexpression of CARMA3 did not affect phosphorylation of TBK1 or IRF3 in RAW264.7 cells, J774A.1 macrophages, and mouse embryonic fibroblast (MEF) cells. In conclusion, *S. aureus* infection induces STING/TBK1/IRF3-mediated IFN-β production in a CARMA3-independent manner.

## 1. Introduction

Bacterial infections pose a serious threat to global public health. *S. aureus*, a gram-positive bacterium, is the etiological pathogen of various diseases in humans and animals. In human beings, it is responsible for a wide variety of infections from skin infection to life-threatening diseases including pneumonia, bacteremia, meningitis, abscesses of various organs, and sepsis [1]. In animals, it causes joint infections and pneumonia in poultry, dermatitis and cellulitis in horses, and mastitis in rabbits and cattle, leading to considerable economic losses [2]. *S. aureus* infections become increasingly complicated for the dissemination of virulent antibiotic-resistant strains such as methicillin-resistant *S. aureus* (MRSA) and multidrug-resistant strains [3].

Type I IFNs play an essential role in host defense [4]. They encompass at least 13 IFN-α (leukocyte IFN) subtypes, IFN-β (fibroblast IFN), IFN-ɛ, IFN-κ, and IFN-ω, all of which bind to IFNα/β receptor 1 (IFNAR1) and IFNAR2 [5]. Production of type I IFNs is rigorously controlled. They are widely and rapidly expressed and participate in immune activation under normal circumstances. Upon sensing cytosolic double-stranded DNA (dsDNA), the endoplasmic reticulum (ER)-resident protein STING relocalizes to the Golgi apparatus and forms the punctate structures, which contain the kinase TBK1, resulting in TBK1 activation and subsequent phosphorylation of IRF3 along with type I INFs production [6].

Although *S. aureus* appears primarily to be an extracellular bacterium, new evidence demonstrated that it can persist intracellularly for different periods of time. *S. aureus* persists locally in the skin and soft tissue. It is able to invade almost any organ once it penetrates the subcutaneous tissues and reaches the blood [7]. Macrophages provide an effective first line of host defense against invasive pathogens. *S. aureus* infection induces production of type I IFNs in lymphocytes and airway epithelial cells [8], and higher mRNA levels of IFN-β in dendritic cells (DCs) [9,10]. CARMA3, a novel scaffold protein, negatively regulates activation of TBK1/IRF3 signaling and resultant IFN-β production in response to single-stranded RNA (ssRNA) virus vesicular stomatitis virus (VSV) or the synthetic double-stranded RNA (dsRNA) analog poly(I:C) in MEFs [11]. In this study, we found that *S. aureus* infection triggers activation of the STING/TBK1/IRF3 pathway and upregulation of CARMA3 at both the mRNA and protein levels, but CARMA3 does not regulate *S. aureus*-induced IFN-β signaling.

## 2. Results

### 2.1. S. aureus Infection Triggers IFN-β Signaling

**Figure 1:**
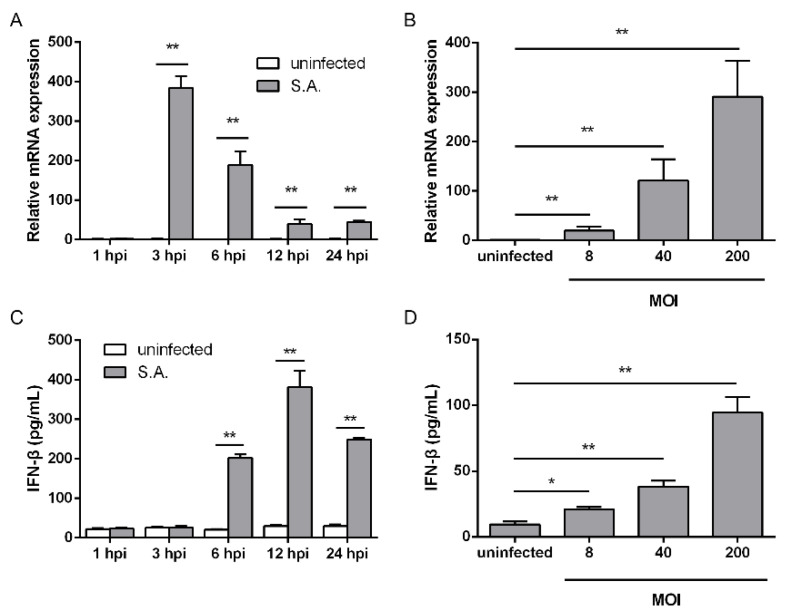
*S. aureus* infection promotes transcription and production of IFN-β. (**A**,**C**) RAW264.7 cells were infected with *S. aureus* for the indicated time at a MOI of 200. (**B**,**D**) Cells were infected with *S. aureus* at the indicated MOI. (**A**,**B**) Cell lysates were subjected to quantitative real-time PCR analysis, normalized to the internal control β-actin. (**C**,**D**) IFN-β levels in the culture supernatant were measured by ELISA assay. Data from one representative experiment of three are presented. * 0.01 < *p* < 0.05, ** *p* < 0.01. Abbreviations: S.A., *S. aureus*.

To test the ability of *S. aureus* to induce IFN-β signaling in macrophages, we initially examined IFN-β mRNA expression at different time points and at various multiplicities of infection (MOIs) in RAW264.7 cells. *S. aureus* infection induced an increased IFN-β mRNA level at three hours post-infection (hpi). The transcriptional levels gradually decreased from 3 to 24 hpi, and similar levels were observed at 12 and 24 hpi (Figure 1A). Following stimulation at various MOIs ranging from eight to 200, IFN-β mRNA expression increased in a dose-dependent manner (Figure 1B). We next sought to determine the levels of IFN-β released into the culture medium. IFN-β levels began to rise at 6 hpi, reached a peak at 12 hpi, and dropped down at 24 hpi following bacterial infection (Figure 1C). *S. aureus*-induced IFN-β production correlated with the MOI ranging from eight to 200 (Figure 1D). These observations indicate that infection with *S. aureus* contributes to the upregulation of IFN-β mRNA expression and IFN-β production in RAW264.7 cells.

### 2.2. S. aureus Induces IFN-β Signaling via the STING/TBK1/IRF3 Pathway

**Figure 2:**
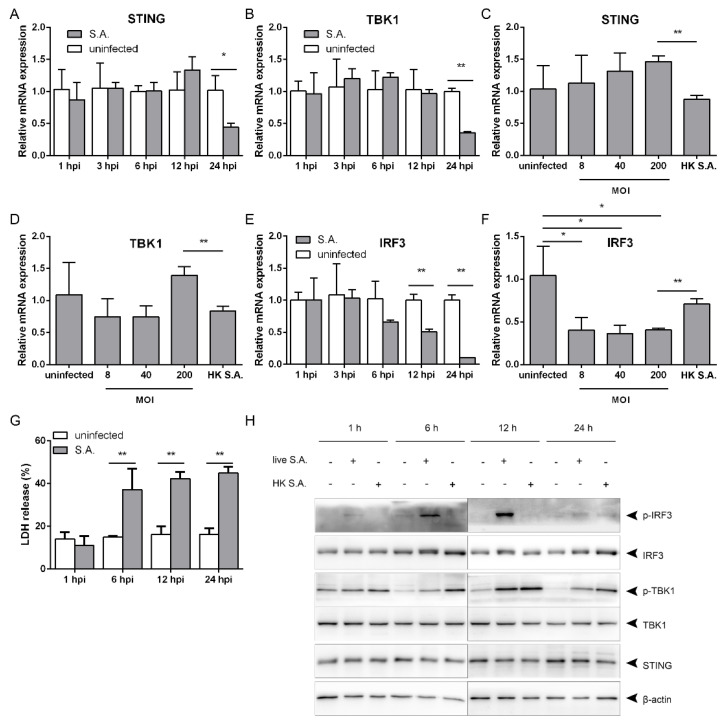
*S. aureus* infection activates the STING/TBK1/IRF3 pathway. (**A**,**B**,**E**,**H**) RAW264.7 cells were infected with *S. aureus* for the indicated time. (**C**,**D**,**F**) Cells were infected with *S. aureus* at the indicated MOI. (**A**,**F**) Cell lysates were subjected to quantitative real-time PCR analysis, normalized to the internal control β-actin. (**G**) Cell lysates were analyzed by immunoblotting. (**G**) Cell death was evaluated by LDH release for the indicated time. * 0.01 < *p* < 0.05, ** *p* < 0.01.Abbreviations: HK S.A., heat-killed *S. aureus* at the MOI of 200.

The STING/TBK1/IRF3 pathway is crucial for regulating type I IFNs production [12]. To clarify whether *S. aureus*-induced IFN-β signaling is dependent on this pathway, we first determined the transcriptional levels. Stimulation with *S. aureus* failed to affect the mRNA levels of STING and TBK1 at a MOI of 200 from 1 to 12 hpi, and led to a decline at 24 hpi (Figure 2A,B). The mRNA levels slightly decreased with no significance following stimulation with the heat-killed bacteria, but were lower than those after infection at a MOI of 200 (Figure 2C,D). IRF3 mRNA levels started to decrease at 12 hpi, and further dropped down at 24 hpi (Figure 2E). The transcriptional levels went down upon bacterial infection at MOIs ranging from eight to 200, and were lower than those upon stimulation with the heat-killed bacteria (Figure 2F). *S. aureus* provokes host cell death during infection [13]. We evaluated cell death by lactate dehydrogenase (LDH) release, and observed that *S. aureus* infection increased LDH release from 6 to 24 hpi (Figure 2G). We next explored the effect of *S. aureus* infection in the STING/TBK1/IRF3 signaling. In contrast to the mRNA levels, the bacterial infection did not influence the protein levels of STING, TBK1, or IRF3 from 1 to 24 hpi (Figure 2H). However, TBK1 was activated at 6 hpi, and IRF3 was activated at 1 hpi, as evidenced by phosphorylation of TBK1 and IRF3, and they remained activated until 24 hpi (Figure 2G). Challenging with heat-killed *S. aureus* caused phosphorylation of TBK1, but had no effect on IRF3 phosphorylation (Figure 2H), indicating that the heat-killed bacteria do not activate IRF3. Taken together, *S. aureus* infection triggers activation of STING/TBK1/IRF3 signaling.

**Figure 3:**
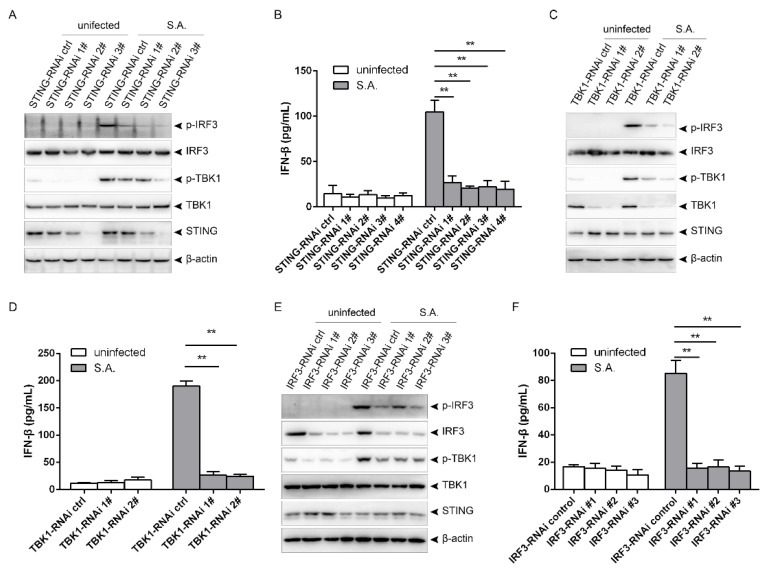
*S. aureus*-induced IFN-β signaling is dependent on the STING/TBK1/IRF3 pathway. (**A**,**B**) RAW264.7 cells were infected with *S. aureus* after STING were silenced using lentivirus-mediated RNA interference (RNAi) targeting to different sequences. (**C**,**D**) Cells were infected with *S. aureus* after TBK1 were silenced. (**E**,**F**) Cells were infected with *S. aureus* after IRF3 were silenced. (**A**,**C**,**E**) Cell lysates were analyzed by immunoblotting. (**B**,**D**,**F**) The levels of IFN-β released in culture supernatant were measured by ELISA assay. ** *p* < 0.01.

To clarify whether *S. aureus*-induced IFN-β signaling is dependent on the STING/TBK1/IRF3 pathway, we detected phosphorylation of TBK1 and IRF3 as well as IFN-β production after lentivirus-mediated silencing of STING, TBK1, or IRF3. Knocking down STING had no effect on protein levels of TBK1 or IRF3, but elicited decreased phosphorylation of TBK1 and IRF3 as well as declines in IFN-β production. The decrease of IRF3 phosphorylation correlated with the levels of STING silencing (Figure 3A,B). Similarly, TBK1 silencing provoked declines in IRF3 phosphorylation and IFN-β production (Figure 3C,D), and knocking down IRF3 decreased TBK1 phosphorylation and IFN-β production (Figure 3E,F). Thus, *S. aureus* infection induces IFN-β production via the STING/TBK1/IRF3 pathway.

### 2.3. S. aureus Induces Upregulation of CARMA3

**Figure 4:**
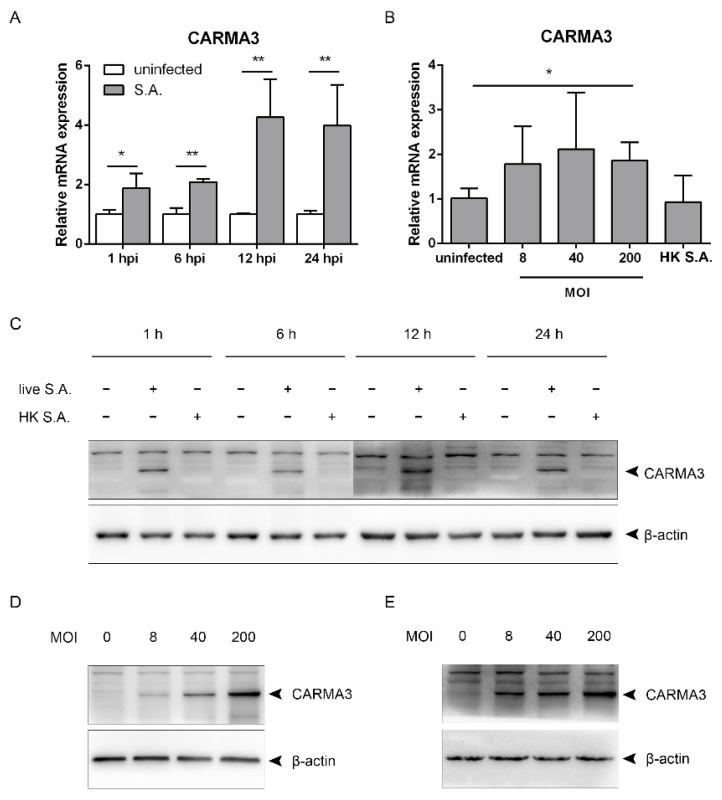
*S. aureus* infection upregulates CARMA3 at both the mRNA and protein levels. (**A**) RAW264.7 macrophages were infected with *S. aureus* for the indicated time. (**B**) RAW264.7 cells were challenged with live or heat-killed *S. aureus* at the indicated MOI. (**C**) RAW264.7 cells were stimulated with live or heat-killed *S. aureus* for the indicated time. (**D**) RAW264.7 cells were infected with *S. aureus* at the indicated MOI. (E) J774A.1 cells were infected with *S. aureus* at the indicated MOI. (**A**,**B**) CARMA3 mRNA levels were assessed using qRT-PCR. (**C**–**E**) Cell lysates were analyzed by immunoblotting. * 0.01 < *p* < 0.05, ** *p* < 0.01.

TBK1/IRF3 activation is negatively regulated by CARMA3 in response to VSV or poly(I:C). In order to explore whether CARMA3 regulates *S. aureus*-induced activation of the STING/TBK1/IRF3 pathway, we first sought to determine the mRNA and protein levels of CARMA3 following the infection. Upon infection at a MOI of 200, the transcriptional levels were elevated at 1 hpi, and continued to be increased at 12 hpi. Similar levels were observed at 12 and 24 hpi (Figure 4A). Infection at the MOI of eight or 40 slightly increased the transcriptional levels at 12 hpi, but with no significance. Stimulation with heat-killed *S. aureus* made no changes compared to the uninfected group (Figure 4B). Infection with live *S. aureus* led to elevated protein levels of CARMA3 from 1 to 24 hpi, while stimulation with heat-killed *S. aureus* did not affect it (Figure 4C). CARMA3 was increased in a dose-dependent manner following infection at MOIs ranging from eight to 200 in RAW264.7 and J774A.1 cells (Figure 4D,E). Collectively, infection with *S. aureus* leads to upregulation of CARMA3 at both the mRNA and protein levels.

### 2.4. S. aureus-Induced Activation of the STING/TBK1/IRF3 Pathway Is Independent of CARMA3

**Figure 5:**
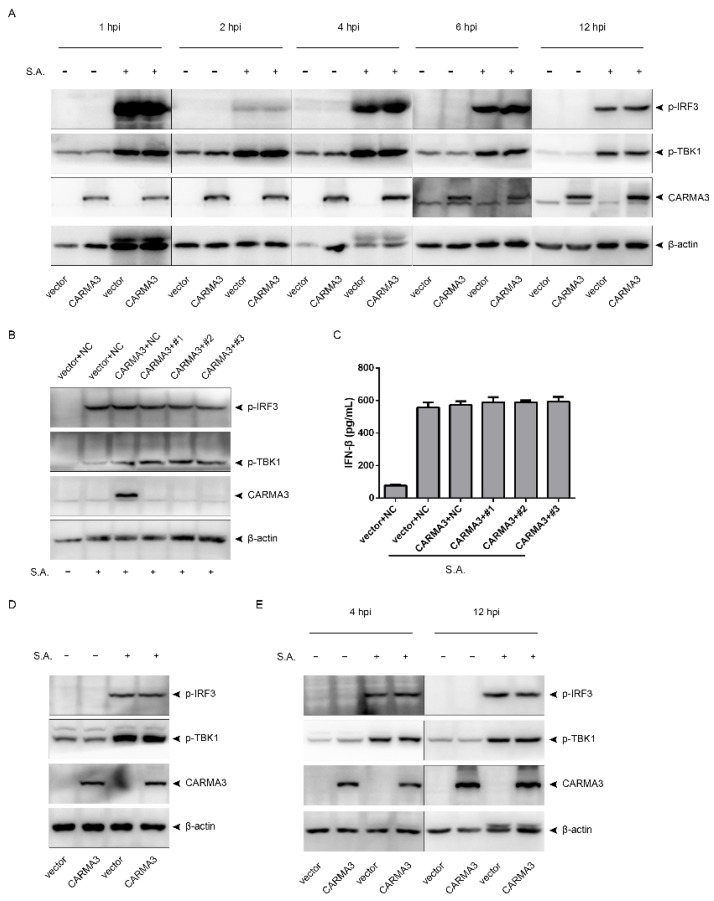
*S. aureus*-induced IFN-β signaling is independent of CARMA3. (**A**) CARMA3 was overexpressed in RAW264.7 cells, and then the cells were infected with *S. aureus* for the indicated time. (**B**,**C**) RAW264.7 cells were transfected with the indicated plasmids, and then were infected with *S. aureus.* (**D**) CARMA3 was overexpressed in J774A.1 cells, and then the cells were infected with *S. aureus.* (**E**) CARMA3 was overexpressed in MEFs, and then the cells were infected with *S. aureus* for the indicated time. (**A**,**B**,**D**,**E**) Cell lysates were analyzed by immunoblotting. (**C**) IFN-β levels in culture supernatant were measured by ELISA assay.

As mentioned above, *S. aureus* infection caused activation of the STING/TBK1/IRF3 pathway and upregulation of CARMA3. We then asked whether CARMA3 regulates activation of the STING/TBK1/IRF3 pathway. RAW264.7 cells were infected with *S. aureus* after CARMA3 was overexpressed. The results showed that overexpression of CARMA3 did not influence phosphorylation of TBK1 or IRF3 from 1 to 12 hpi (Figure 5A). Knocking down CARMA3 failed to change *S. aureus*-induced phosphorylation of TBK1 and IRF3 in CARMA3-overexpressing RAW264.7 cells (Figure 5B). Accordingly, overexpression or silencing of CARMA3 did not change IFN-β production (Figure 5C). To rule out the possibility that this CARMA3-independent activation of the STING/TBK1/IRF3 pathway was associated with the cell type, we infected J774A.1 cells and MEFs in which CARMA3 were overexpressed with *S. aureus*. Consistently, overexpression of CARMA3 did not affect phosphorylation of TBK or IRF3 in these two cell lines (Figure 5D,E). Together, *S. aureus* infection induces activation of the STING/TBK1/IRF3 pathway and IFN-β release in a CARMA3-independent manner.

### 2.5. S. aureus-Induced Activation of the STING/TBK1/IRF3 Pathway Is Independent of B Cell Lymphoma 10 (BCL10)

**Figure 6:**
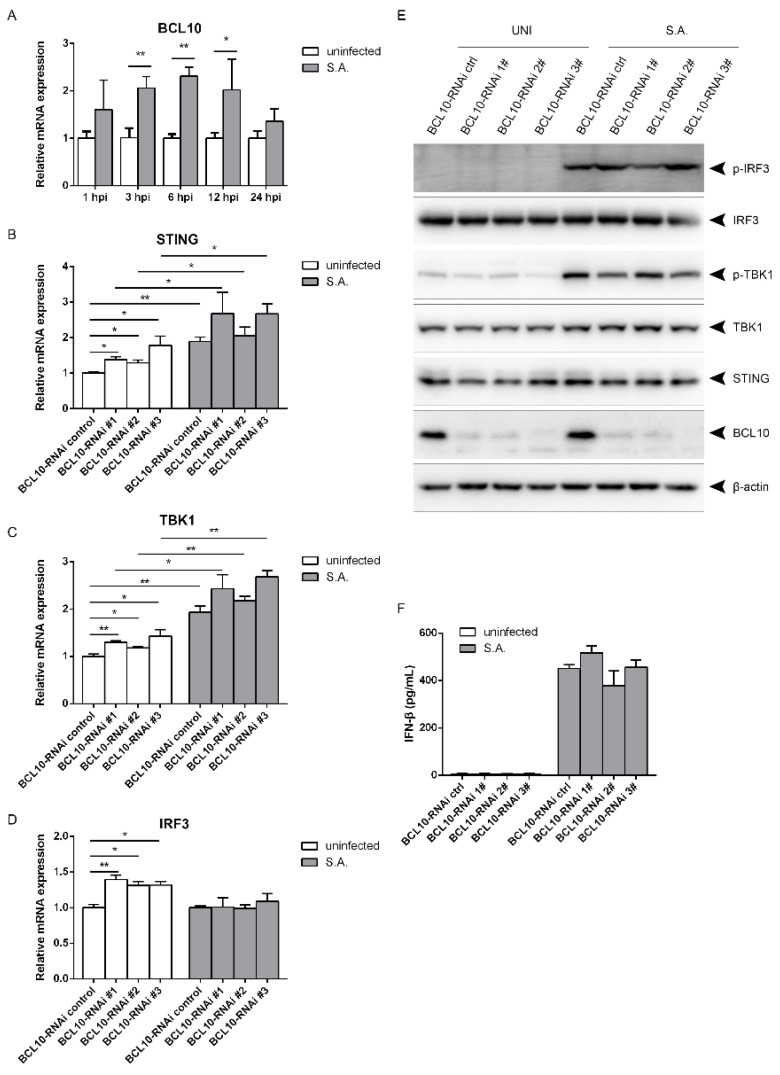
*S. aureus*-induced IFN-β signaling is independent of BCL10. (**A**) RAW264.7 cells were infected with *S. aureus* for the indicated time. (**B**–**F**) BCL10 were silenced, and then the cells were infected with *S. aureus*. (**A**–**D**) Cell lysates were subjected to quantitative real-time PCR analysis, normalized to the internal control β-actin. (**E**) Cell lysates were analyzed by immunoblotting. (**F**) IFN-β levels in culture supernatant were measured by ELISA assay. * 0.01 < *p* < 0.05, ** *p* < 0.01.

BCL10 plays a similar function as CARMA3 in regulation of IFN-β signaling in response to VSV or poly(I:C) [11]. We wanted to determine whether BCL10 affects activation of the STING/TBK1/IRF3 pathway. The transcriptional levels began to increase at 3 hpi, and remained elevated until 12 hpi following *S. aureus* infection (Figure 6A). The mRNA levels of STING, TBK1, and IRF3 were increased by less than two-fold after silencing of BCL10 in the resting state. Knocking down BCL10 led to higher transcriptional levels of STING and TBK1, but not of IRF3 upon the bacterial infection (Figure 6B–D). Silencing of BCL10 did not influence the protein levels of STING, TBK1, or IRF3. *S. aureus* infection failed to affect phosphorylation of TBK1 and IRF3 as well as IFN-β production (Figure 6E,F), suggesting that BCL10 does not regulate *S. aureus*-induced activation of the STING/TBK1/IRF3 pathway and the subsequent IFN-β production.

## 3. Discussion

*S. aureus* can invade various types of non-professional and professional phagocytes, and is able to survive engulfment by professional phagocytes such as macrophages [14,15]. Many reports have demonstrated that *S. aureus* can persist intracellularly [7,16], and utilizes the intracellular environment as a critical refuge for survival and dissemination in the hosts via manipulation of innate immunity, including autophagy [17] and apoptosis [18]. Toll-like receptor 2 (TLR2) favors its intracellular survival in THP-1 monocytes [19]. Type I IFNs signaling serves as a vital component of innate immune response to virus and intracellular bacteria. Ifnar knockout markedly increases the bacterial load in the spleen at four hours after inoculation, and slightly increases the bacterial burden at 24 h after inoculation with no significance following intranasally infected with epidemic MRSA USA300. However, more Ifnar^−/−^ mice succumbed to infection due to lethal pneumonia compared to WT mice [8]. *S. aureus* infection induces TBK1/IRF3 axis activation and production of type I IFNs in lymphocytes, DCs [9,10], and airway epithelial cells [8]. Here, we showed that *S. aureus* infection induces STING/TBK1/IRF3 axis activation and IFN-β release in murine macrophages.

*S. aureus* infection influenced phosphorylation of TBK1 and IRF3 rather than protein levels of TBK1 and IRF3, indicating that the bacterial infection initiates IFN-β signaling via controlling the activities of these two proteins by posttranslational modification. This is also observed for other agonists, including lipopolysaccharide (LPS) [20], VSV [21], Sendai virus [22], and poly(I:C) [23,24]. *S. aureus* infection caused phosphorylation of TBK1 and IRF3, activating the STING/TBK1/IRF3 pathway. This led to upregulation of IFN-β at the mRNA level at 3 hpi and at the protein level at 6 hpi. The increase of IFN-β decreased the transcriptional levels of STING, TBK1, and IRF3 at different time points. Of these three genes, IRF3 gene is the most sensitive. The RNA level began to drop down slightly with no significance at 6 hpi, and continued to decrease at 12 and 24 hpi. The RNA levels of TBK1 and STING declined at 24 hpi. Several mediators of negative feedback mechanism of type I IFN have been reported, including interferon-stimulated gene 56 (ISG56) [25], the E3 ubiquitin ligase RBCC protein interacting with PKC1 (RBCK1) [26], interferon-induced transmembrane protein 3 (IFITM3) [27], and FoxO1 (Forkhead box protein O1) [28].

IFN-β is released later than it is transcribed, which may be caused by delay in protein synthesis and secretion [29]. This delay is also observed in hypoxia-induced osteoprotegerin [30] and sphingosine 1-phosphate-induced IL-18 [31]. In this study, transcriptional levels of CARMA3 at 1 and 6 hpi, also at 12 and 24 hpi, are similar following infection. The mechanism needs to be explored.

Upon nonspecific binding to cytosolic DNA, cyclic GMP-AMP (cGAMP) synthase (cGAS) is activated to catalyze the synthesis of cGAMP from ATP and GTP. cGAMP binds STING, inducing an active STING conformation. Activated STING then recruits TBK1 to trigger phosphorylation of IRF3 and type I IFN production [32,33]. cGAS is further induced by IFN-β through two adjacent IFN-sensitive response elements in the promoter region of cGAS [34], subsequently promoting phosphorylation of TBK1 and IRF3. Here, silencing of IRF3 decreased production of IFN-β, which caused reduced protein level of phosphorylated TBK1, although IRF3 functions downstream of TBK1.

*S. aureus*-induced production of type I IFNs is associated with the bacterial autolysis. Compared to USA300 strains, the strain 502A is more autolytic, and provides more increased pathogen-associated molecular patterns (PAMPs), such as peptidoglycan [9] and CpG DNA, which is sensed by TLR9 [10], thus leading to a stronger induction of type I IFN signal transduction. Heat-killed 502A induces less IFN-β mRNA expression than live bacteria in DCs, while USA300 has no difference when heat inactivated [9,10]. In this study, heat-killed *S. aureus* failed to trigger IRF3 activation, yet the level of phosphorylated TBK1 induced by the heat-killed bacteria was similar to that by the live bacteria.CARMA3 recruits BCL10 and MALT1 to generate the CBM (CARMA3/BCL10/MALT1) complex, which subsequently functions to influence the downstream signaling pathways [35]. CARMA3 and BCL10 play a similar role in type I IFNs signaling via the STING/TBK1/IRF3 pathway and inflammatory responses via the NF-κB pathway in response to ssRNA viruses or the synthetic dsRNA analog. However, MALT1 does not play a critical role in these pathways [11]. CARMA3 prevents mitochondrial antiviral-signaling protein (MAVS) from assembling high-molecular-weight aggregates, subsequently restricting activation of the STING/TBK1/IRF3 pathway [11]. Poly(I:C) stimulation triggers NF-κB activation as early as 0.5 hpi [36,37]. CARMA3 is degraded via ubiquitination-proteasome pathway after NF-κB activation upon stimulation with poly(I:C) or VSV, leading to release and oligomerization of MAVS as well as subsequent activation of the STING/TBK1/IRF3 pathway. This starts to occur at approximately 4 hpi in MEFs [11]. Our study showed that *S. aureus* infection triggered upregulation of CARMA3 expression at both the mRNA and protein levels from 1 to 24 hpi in RAW264.7 cells. The protein level was also increased in J774A.1 cells. However, *S. aureus*-induced activation of the STING/TBK1/IRF3 pathway is independent of CARMA3. CARMA3 overexpression did not affect phosphorylation of TBK1 or IRF3 in RAW264.7 cells from 1 to 12 hpi. *S. aureus* may manipulate the ubiquitination-proteasome-mediated degradation of CARMA3. VSV induces degradation of exogenous CARMA3, and the protein level drops down markedly at 3 hpi [11]. However, *S. aureus* infection did not lead to decreased CARMA3 expression in RAW264.7 cells or MEFs even at 12 hpi.

Upon stimulation with virus RNA, retinoic acid-inducible gene 1 (RIG-I), a cytosolic RNA sensor, undergoes conformational changes and binds MAVS, leading to activation of the TBK1/IRF3 pathway via interaction between MAVS and TBK1 [38]. CARMA3 binds MAVS to regulate this pathway [11]. STING is dispensable for RNA virus-induced pro-duction of type I IFNs, although it is necessary to restrict the replication [39]. As for *S. aureus*, STING is required to induce type I IFNs signaling, and no evidence demonstrated that the bacteria is able to activate RIG-I and MAVS. CARMA3-independent regulation of IFN-β production triggered by S. aureus may be associated with RIG-I and MAVS, but it still needs to be tested.

**Figure 7:**
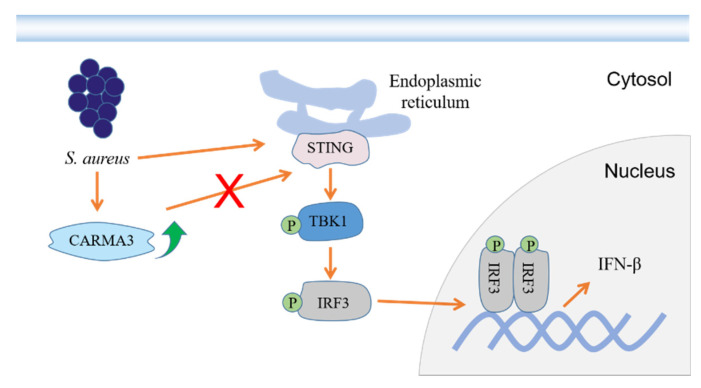
*S. aureus* induces IFN-β signaling via a CARMA3-independent mechanism. *S. aureus* infection contributes to phosphorylation of TBK1 and IRF3 and resultant IFN-β production. CARMA3 expression at both the mRNA and protein levels is increased, but fails to regulate STING/TBK1/IRF3-mediated IFN-β production.

In conclusion, *S. aureus* infection induces STING/TBK1/IRF3-mediated IFN-β production via a CARMA3-independent mechanism (Figure 7). Our study contributes to a better understanding of the regulation of type I IFN response to infection with *S. aureus*.

## 4. Materials and Methods

### 4.1. Reagents and Antibodies

The following reagents and antibodies were purchased from the indicated suppliers: anti-TBK1 and anti-p-TBK1 antibodies from Abcam, Cambridge, MA, USA; anti-STING, anti-β-actin, anti-IRF3, and anti-STING antibodies from Proteintech, Wuchan, China; anti-CARMA3 and anti-p-IRF3 antibodies from ABclonal, Wuhan, China.

### 4.2. Bacteria and Cell Culture

*S. aureus* ATCC 29213 was purchased from Hangzhou Tianhe Microorganism Reagent Co., Ltd. (Hangzhou, Zhejiang, China), and cultured in Mueller–Hinton Broth (MHB, Qingdao Hope Biol-Technology Co., Ltd., Qingdao, Shandong, China) at 37 °C. RAW264.7, J774A.1, and MEF cells were obtained from Xiehe Medical University (Beijing, China), and maintained in DMEM (Gibco, Grand Island, NY, USA) containing 10% fetal bovine serum (FBS, PAN Biotech, Aidenbach, Germany). Bacteria were subcultured at a 1:50 dilution of an overnight culture for 2.5 h in MHB at 37 °C under shaking. Cells were challenged at the indicated MOI for 30 min, then washed three times with preheated PBS, and fresh culture medium supplemented with 100 µg/mL of gentamycin was added to kill extracellular bacteria. Samples were harvested at the indicated time. All experiments were performed three times.

### 4.3. Western Blotting

Western blot analysis was performed as previously described [40]. Briefly, an equal amount of samples was loaded onto SDS-PAGE, and proteins were transferred onto PVDF membranes. Membranes were blocked with 5% non-fat milk in TBS, then incubated with the corresponding primary antibodies overnight at 4 °C and the appropriate HRP-labeled secondary antibody for 50 min at 37 °C, and finally developed using an enhanced chemiluminescence (ECL) detection kit (Millipore, Burlington, MA, USA).

### 4.4. Quantitative Real-Time PCR

Total RNA extraction was performed using Total RNA Extraction Kit (Solarbio, Beijing, China), and reverse transcription was performed using HiScript II Q RT SuperMix for qPCR (Vazyme, Nanjing, Jiangsu, China). Quantitative PCR was carried out in a Bio-Rad CFX960 using AceQ qPCR SYBR Green Master Mix (Vazyme). Primers were obtained from GENEWIZ, Suzhou, China (Table 1). PCR protocol was: 95 °C for 30 s and 40 two-step cycles: 95 °C for 10 s and 60 °C for 35 s.

### 4.5. IFN-β ELISA

ELISA for IFN-β was performed using Mouse IFN-β ELISA KIT (Solarbio, Beijing, China) according to the manufacturers’ instructions.

### 4.6. Lentivirus-Medited RNAi

Lentivirus-mediated RNAi was performed utilizing the CRISPR-Cas9 system [41]. Lentiviral vector plasmid lentiCRISPR v2, lentiCRISPR v2 harboring non-targeting sequences (NC), packaging plasmid pGag-pol, and envelope plasmid pVSV-G were generously provided by Hongbing Shu of Wuhan University, China. Recombinant lentiviruses were prepared by co-transfecting HEK293T cells with lentiCRISPR v2 harboring the double-stranded oligonucleotides corresponding to the target sequences (Table 2) plus pGag-pol and pVSV-G plasmids. RAW264.7 cells were infected with the viruses harvested two days after transfection. The infected cells were selected using puromycin for at least five days.

### 4.7. Plasmid Transfection

LentiCRISPR v2 harboring CARMA3-targeting sequences (Table 3) and pLOV-CARMA3 were transfected using Lipofectamine 3000 (Thermo Fisher Scientific, Waltham, MA, USA) according to the manufacturers’ instructions. 

### 4.8. LDH Release Assay

LDH release was measured using LDH Cytotoxicity Assay Kit (Cayman Incorporated, Ann Arbor, MI, USA) according to the manufacturer’s instructions.

### 4.9. Statistical Analysis

All assays were performed in three independent experiments, and data were analyzed using GraphPad Prism 5.0 software and Student’s *t* test; *p* < 0.05 values were considered statistically significant.

## Figures and Tables

**Table 1:** Primers used for quantitative real-time PCR.

Gene	Forward Primer (5′–3′)	Reverse Primer (5′–3′)
IFN-β	GCACTGGGTGGAATGAGACT	AGTGGAGAGCAGTTGAGGACA
CARMA3	AGGCAGGAGTGGTTCTGTACT	TCTTCAGGTTGCTTCGAGGAC
STING	TGGCTGCTGATGCCATACTC	CACAGCTCTTCAGCCAGACA
TBK1	ACACATGACGGCGCATAAGA	CGGCTCGTGACAAAGATAGGA
IRF3	ACAGATGGCTGACTTTGGCA	GCAGCTAACCGCAACACTTC
BCL10	GAGCATCCACTGTCATGTACCA	AGGAAGAGTGGCTGAAGAGAA
β-actin	CAGAGCAAGAGAGGTATCCTGAC	AAGGTCTCAAACATGATCTGGGT

**Table 2:** Target sequences for CRISPR-Cas9 system.

Name	Sequence (Sense, Antisense)
STING-targeting sequences	
Target sequence 1:	CACCGTTAGAGGAATTCGGAGTGCG
	AAACCGCACTCCGAATTCCTCTAAC
Target sequence 2:	CACCGCGCACTCCGAATTCCTCTAA
	AAACTTAGAGGAATTCGGAGTGCGC
Target sequence 3:	CACCGAGCGGTGACCTCTGGGCCGT
	AAACACGGCCCAGAGGTCACCGCTC
Target sequence 4:	CACCGTGTTGGGGTCAACTACACTC
	AAACGAGTGTAGTTGACCCCAACAC
TBK1-targeting sequences	
Target sequence 1:	CACCGCATAAGCTTCCTTCGCCCAG
	AAACCTGGGCGAAGGAAGCTTATGC
Target sequence 2:	CACCGGAGGAGCCGTCCAATGCGTA
	AAACTACGCATTGGACGGCTCCTCC
IRF3-targeting sequences	
Target sequence 1:	CACCGACCAGCCAGGGCAAAATCCG
	AAACCGGATTTTGCCCTGGCTGGTC
Target sequence 2:	CACCGGAACGAGGTTCAGGATCCCG
	AAACCGGGATCCTGAACCTCGTTCC
Target sequence 3:	CACCGCCAGTGGTGCCTACACCCCG
	AAACCGGGGTGTAGGCACCACTGGC
BCL10-targeting sequences	
Target sequence 1:	CACCGCACTGTCATGTACCACCCGG
	AAACCCGGGTGGTACATGACAGTGC
Target sequence 2:	CACCGCCGAACTTCAAGTAGAAAAC
	AAACGTTTTCTACTTGAAGTTCGGC
Target sequence 3:	CACCGCGCACCGTCCCTCACGGAGG
	AAACCCTCCGTGAGGGACGGTGCGC

**Table 3:** CARMA3-targeting sequences.

Name	Sequence (Sense, Antisense)
Target sequence 1:	CACCGACGGCTCCGGAGGCGCGACG
	AAACCGTCGCGCCTCCGGAGCCGTC
Target sequence 2:	CACCGCGGTACGGTTAGCGCGGCAC
	AAACGTGCCGCGCTAACCGTACCGC
Target sequence 3:	CACCGTGGCGGCGGGCTCACCGGTA
	AAACTACCGGTGAGCCCGCCGCCAC

## Data Availability

The data presented in this study are available on request from the corresponding author.
